# Dyadic Profiles of Couples Coping With Body Image Concerns After Breast Cancer: Preliminary Results of a Cluster Analysis

**DOI:** 10.3389/fpsyg.2022.869905

**Published:** 2022-03-23

**Authors:** Emanuela Saita, Giulia Ferraris, Chiara Acquati, Sara Molgora, Antonia Sorge, Francesco Valenti, Massimo Maria Grassi, Denise Vagnini

**Affiliations:** ^1^Department of Psychology, Catholic University of Sacred Heart, Milan, Italy; ^2^Department of Health Psychology, University Medical Center Groningen, University of Groningen, Groningen, Netherlands; ^3^Graduate College of Social Work, University of Houston, Houston, TX, United States; ^4^Department of Clinical Sciences, College of Medicine, University of Houston, Houston, TX, United States; ^5^Department of Health Disparities Research, The UT MD Anderson Cancer Center, Houston, TX, United States; ^6^Breast Unit, Humanitas Gavazzeni Clinical Institute, Bergamo, Italy

**Keywords:** body image, breast cancer, couples, emotional wellbeing, relationship functioning

## Abstract

Breast cancer treatments have multiple adverse effects, including concerns about body appearance and function that are experienced by most patients. Altered body image negatively affects mental health, social, and relationship functioning. While the relationship with a partner is critical for patients’ psychological wellbeing and partners can promote positive body image, limited research has investigated individual and relational factors affecting the experience of both. This cross-sectional study aimed at (1) exploring rates of body image concerns among breast cancer patients, and (2) identifying dyadic profiles among participating dyads. Couples composed by patients who had undergone surgery and their romantic partners (*n* = 32) were recruited from the Breast Unit of a hospital in northern Italy. Both partners completed measures of personality characteristics (BFQ-2), psychological distress (HADS), coping flexibility (PACT), dyadic coping (DCQ), and closeness (IOS). Body image (BIS) and adjustment to cancer (Mini-MAC) measures were completed by patients only. K-mean cluster analyses identified 2-cluster solution among patients and partners, respectively. “Active patients” (cluster-1) reported low rates of body image concerns (*p* < 0.001), anxious preoccupation, negative dyadic coping, and self-oriented stress communication (*p* < 0.05), compared to “worried patients” (cluster-2). “Comfortable partners” (cluster-1) reported lower anxiety and depression (*p* < 0.001), self-oriented negative dyadic coping and closeness (*p* < 0.05) than “uncomfortable partners” (cluster-2). Three different dyadic profiles emerged: *functional*, *dysfunctional*, and *ambivalent*. Significant variations (*p* < 0.05) by anxiety, depression, and delegating dyadic coping existed. Results indicate there are groups of couples at greater risk for impaired psychological distress and body image concerns, which should be addressed in the context of dyadic psychosocial interventions.

## Introduction

A breast cancer (BC) diagnosis is an unexpected and destabilizing event that can have a potentially negative impact on quality of life over time (Zimmermann et al., 2010). Among the long-term negative consequences of the disease and its treatments (i.e., surgery, neoadjuvant/adjuvant therapies) impaired physical functioning, femininity, and sexual health have been extensively documented. Impacts in these areas may also produce a negative body image, which is defined as perceptions, thoughts, or emotions about one’s physical appearance (Cash, 2004; Fobair et al., 2006; Lindwall and Bergbom, 2009; Falk Dahl et al., 2010). Between 17–33% of BC patients and 15–30% of long-term survivors report some degree of body image concerns due to irreversible (e.g., scarring/amputations) or temporary (e.g., hair loss, weight, and hormonal fluctuations) changes in appearance (Begovic-Juhant et al., 2012; McKean et al., 2013; Fingeret et al., 2014).

Body image concerns have been linked to compromised psychological functioning (e.g., mood disturbances and severe depressive symptoms) especially among patients who undergone invasive treatments (Moreira and Canavarro, 2010; Morales-Sánchez et al., 2021). Previous studies have primarily focused on body image as an aspect of individuals’ psychosocial adjustment (Rowland and Metcalfe, 2014) or as a predictor of anxiety and depression (Falk Dahl et al., 2010), and have considered the effects of surgery (Fingeret et al., 2013) and age (Rosenberg et al., 2013; Leigh et al., 2019). Women undergoing more radical surgery approaches reported significantly worse adaptation, and younger women report greater distress for this domain of quality of life (Acquati and Kayser, 2019; Davis et al., 2020). Personality characteristics also influence the psychosocial adjustment to cancer, with different levels of flexibility associated with specific personality traits. For example, extraversion and conscientiousness predicted more problem-solving and cognitive restructuring, compared to neuroticism (Connor-Smith and Flachsbart, 2007). Similarly, personality traits correlated with relationship functioning, highlighting that couples characterized by higher levels of neuroticism experienced lower levels of marital satisfaction. On the contrary, couples reporting high conscientiousness were more satisfied (Sayehmiri et al., 2020). In addition, the quality of patients’ relationship with a partner is also a crucial factor facing cancer (Zimmermann et al., 2010; Shrout et al., 2020). Empirical evidence linked supportive and satisfactory relationships with positive body image in the immediate post-operative period (2–6 weeks), as well as 1 year later (Brandão et al., 2017; Cairo Notari et al., 2017; Saita et al., 2018).

However, research on couple-level factors, such as dyadic coping and relationship satisfaction, is scarce. Due to the interdependence that exists among patients and partners, both can be profoundly affected by the cancer experience (Kayser et al., 2007; Zimmermann, 2015). Couples experienced several changes to their previous roles within the dyad, with marital adjustment contributing to patient’s physical, mental, and sexual functioning. Moreover, relationship characteristics have been linked to the level of burden experienced (Keesing et al., 2016; Brandão et al., 2017). Dyadic coping behaviors may be particularly salient for patients’ body image, since women’s self-image is established, in part, within the context of their intimate relationships (Scott et al., 2004). For instance, the Michelangelo Phenomenon (which refers to how our self-image is constructed according to how our partner sees us, in the same way Michelangelo saw the sculpture hidden in the stone) contributes to illustrate the influence of partners’ responses on patients’ body image (Drigotas et al., 1999). Recent studies have documented that partners’ empathic responses moderated the association between patients’ body image concerns and depressive symptoms after surgery, while partners’ disgusting responses were correlated with patients’ self- reported feelings of disgust (Fang et al., 2015; Azlan et al., 2017).

These emerging results confirm that, in addition to the above-mentioned individual variables, the quality of close relationships and the interaction between partners might protect couples from negative outcomes both at the individual and relational level (Manne and Badr, 2010; Saita et al., 2015; Kayser and Acquati, 2019). Patients and partners mutually influence each other in their stress and coping process, confirming that the experience of cancer is influenced by the patients’ interpersonal context (Hagedoorn et al., 2008). For instance, the ability to display relational mutuality promotes adaptive dyadic coping behaviors (Acquati and Kayser, 2019; Kayser and Acquati, 2019). Therefore, coping with cancer-related body image concerns should be regarded as a dyadic affair, and investigated as a stressor regarding both partners.

The aim of the study is to (1) explore rates of body image concerns among BC patients 1 week after surgery, and (2) identify dyadic profiles of couples according to individual and relational variables. We assumed a relational perspective, guided by the *Systemic-Transactional Model of Dyadic Coping* (STM) by Bodenmann (1995). This model assumes that the mechanism of stress and coping is a social process of interdependence between two partners. A threatening event affects both individuals’ psychological wellbeing and the couple as a unit. Stress is conceived as a we-stress, and the disease is represented as a we-disease (Kayser et al., 2007). A good dyadic functioning consists in responding to the problem of both by providing mutual support, with the aim of re-establishing the homeostatic balance of the dyad (Bodenmann, 1995, 1997, 2005). Therefore, in this study it was hypothesized that:

(1) Patient’s self-reported body image perceptions is one of the pivotal variables characterizing dyadic profiles.
(2) Both individual and relational factors influence the psychosocial experience of patients and partners, and they contribute in profiling couples facing BC.
(3) Dyadic profiles will distinguish between functioning vs. burdened couples.

## Methods

### Procedure and Participants

A cross-sectional survey of 32 couples composed by BC patients and their partners (*N* = 64) was conducted in 2018–2019. Subsequently, data collection was interrupted due to COVID-19 pandemic. In addition, considering the effects of the pandemic on cancer patients and caregivers’ psychosocial wellbeing (Dhada et al., 2021; Ludwigson et al., 2022), couples recruited after the first lockdown were considered intrinsically different and therefore were not included in the present contribution.

Participants were recruited from the Breast Unit of a Hospital located in Northern Italy using a convenience, non-probabilistic sampling approach. They were invited to participate in the research study by the medical staff (e.g., surgeons or nurses) the day after the surgery or during the patients’ follow-up visit (1 week later). The same day, interested participants met with trained members of the research team and psycho-oncologists to complete printed copies of the survey. Patients were eligible to participate if they: (1) were ≥ 18 years, (2) had received a diagnosis of BC within the previous 6 weeks, (3) had surgery (i.e., quadrantectomy or mastectomy), (4) were in a romantic relationship with a partner available to participate, and (5) were Italian speaking. Eligible partners: (1) had to be ≥ 18 and (2) Italian speaking. Exclusion criteria for both comprised having a declared serious mental illness or dementia symptoms. Couples provided informed consent before survey completion. Participants provided socio-demographic information (e.g., sex, age, marital status, and rural/urban location), while clinical information about surgery (i.e., quadrantectomy or mastectomy) was obtained medical records. Data were anonymized through an alphanumeric code, identical for members of the dyad to match partners. All procedures were approved by the Ethics Committee of the participating institutions.

### Measures

#### Individual Variables

##### Body Image

The Italian version of Body Image Scale (BIS) was used (α = 0.93) (Hopwood et al., 2001; Cheli et al., 2016). It consists of a 10-item questionnaire assessing diverse dimensions of body image in cancer patients after surgery, or treatment (example item: Have you felt less physically attractive as a result of your disease or treatment?). Items are scored on a 4-point Likert response scale (from 0 = not at all to 3 = very much) and the final score range 0–30, with higher scores corresponding to more perceived body image concerns. The literature does not provide intermediate cut-offs for the interpretation of clinical aspects. For this reason, total scores were organized in three categories according to previous studies conducted by the team of investigators (e.g., Saita et al., 2018): “good body image” (0–10), “composite body image” (11–20), and “impaired body image” (21–30).

##### Coping With Cancer

The Mental Adjustment to Cancer (Mini-MAC) (Watson et al., 1988; Grassi et al., 2005) is a 29-item questionnaire (0.78 < α < 0.93). Respondents rate on a 4-point Likert scale (from 1 = completely disagree; to 4 = completely agree) the prevailing coping style used to cope with cancer: Fighting Spirit (example item: I am determined to beat this disease); Hopeless/Helplessness (example item: I feel like giving up); Anxious Preoccupation (example item: I feel very angry about what has happened to me); Fatalism (example item: At the moment I take one day at a time); and (5) Avoidance (example item: I distract myself when thoughts about my illness come into my head).

##### Personality Traits

The Italian version of the Big Five Questionnaire (BFQ-2) Short Form (0.60 < α < 0.90) (Caprara et al., 1993) presents fifteen personality characteristics (e.g., effusive; unselfish; creative), ranging on a 7-points Likert scale (from 1 = it does not describe me at all; to 7 = it describes me perfectly). It was administered to define five dimensions of personality: extraversion; agreeableness; conscientiousness; openness; and neuroticism.

##### Coping Flexibility

The Italian version of Perceived Ability to Cope with Trauma (PACT) Scale (Bonanno and Pat-Horenczyk, 2011; Saita et al., 2017), consisting of 20 items scored on a 7-step Likert scale (from 1 = not capable at all; to 7 = extremely capable), was used to assess the perceived ability of processing the trauma (Trauma Focus Subscale α = 0.91; example item: I reflect on the meaning of the event), and moving beyond the trauma (Forward Focus Subscale α = 0.79; example item: I remind myself that things will get better). The Flexibility score, which indicates the ability to modify coping strategies depending on the environment/social context, is obtained by combining the sum and the discrepancy score.

##### Psychological Distress

The Italian version of the Hospital Anxiety and Depression Scale (HADS) (Zigmond and Snaith, 1983; Costantini et al., 1999) evaluated distress, anxiety, and depression. It contains two 7-item Likert scales ranged 0–3 measuring respectively anxiety (HADS-A 0.68 < α < 0.93; example item: Worrying thoughts go through my mind), and depression (HADS-D 0.67 < α < 0.90; example item: I feel as if I am slowed down). The total score of both subscales range 0–21. The cut-off of ≥ 11 defines the presence of psychological morbidity with “abnormal” level of mood disturbances, while scores of 8–10 are indicative of a “borderline” level, and 0–7 scores characterize “normal” profiles.

#### Relational Variables

##### Dyadic Coping

The Italian version of the Dyadic Coping Questionnaire (DCQ) (Bodenmann, 2005; Donato et al., 2009) is a 37-item measure assessing different dyadic coping styles (0.72 < α < 0.81). Items are scored on a 5-point Liked scale ranging from 1 (very rarely) to 5 (very often). Example of items are: We try to manage the problem together and find concrete solutions; When I am too busy, my partner helps me out; When he/she is stressed I avoid him/her. The inventory includes the following subscales: common dyadic coping, supportive, delegating, and negative. In addition, DCQ includes subscales for stress communication, and two single items concerning satisfaction with and efficiency of dyadic coping. Except for the two single items and common dyadic coping, all subscales measure the respondents’ own behavior (self-measured) and the respondents’ perception of their partner’s behavior (other-measured).

##### Interpersonal Closeness

The Inclusion of the Other in the Self Scale (IOS) (Aron et al., 1992), a single item on a 7-point Likert scale (from 1 = absence of closeness, to 7 = extreme closeness), was used to measure the degree of closeness, or intersubjectivity with the partner. It is composed of seven Euler-Venn diagrams; the first set of each diagram represents the Self while the second represents the significant Other (i.e., the romantic partner). The width of the intersection between the two sets indicates the degree of proximity within the couple. From a graphic point of view, the amplitude of diagrams’ intersection increases linearly: the first pair shows an absence of perceived closeness, while the seventh shows an almost total overlap.

### Data Analysis

Data were analyzed using IBM SPSS Statistical Software version 27.0 (IBM Corp., 2020). Participants (*n* = 32 patients, *n* = 32 partners) completed the background information sheet with socio-demographic data and the survey with psychological measures. All metric variables were assessed to verify normal distribution for asymmetry and kurtosis (George and Mallery, 2010). Missing values analysis (MVA) showed no missing data. Descriptive statistics (i.e., frequency, mean, and SD) and *t*-test (*p* < 0.05) for independent samples were performed to present psychological variables by role (patients vs. partners). Then, two separated *k-mean cluster analyses* were conducted on patients and partners’ subsamples to identify groups characterized by high between-clusters homogeneity, and high between-clusters distance (Hair and Black, 2000; Henry et al., 2005). All the individual and relational variables assessed in the study were included. Given the exploratory nature of the study, different groupings were tested. Each cluster was then labeled based on participants’ prevailing psychological characteristics. Output tables demonstrated the belonging of each statistical unit (i.e., each subject) to the separately extracted clusters. Then, each couple was examined to identify whether (1) partners belonged to a cluster of greater individual and relational wellbeing; (2) both partners belonged to a cluster of impaired individual and relational wellbeing; and (3) one partner belonged to a cluster of greater wellbeing and the other to a cluster of more impaired wellbeing. Descriptive analysis of the resulting dyadic profiles was performed. Finally, individual and relational variables were included as dependent variables in a univariate one-way analysis of variance (ANOVA), with Bonferroni correction in *post hoc* tests, to investigate dissimilarities between couples’ profiles.

## Results

Participants’ demographics and patients’ clinical characteristics are summarized in Table 1.

**TABLE 1:** Participants’ demographics and clinical characteristics.

Sociodemographic and clinical variables	Patients (*n* = 32)	Partners (*n* = 32)
**Sex**		
Females	32 (100%)	0
Males	0	32 (100%)
**Living situation**		
Cohabitation	32 (100%)	32 (100%)
Age M ± SD (range)	60.19 ± 10.72 (35–75)	61.09 ± 10.73 (35–77)
**Surgery**		
Conservative surgery	21 (65.6%)	NA^a^
Mastectomy	11 (34.4%)	NA^a^
**Body Image**		
Positive body image (BIS score 0–10)	26 (81.3%)	NA^a^
Composite body image score (BIS score 11–20)	4 (12.5%)	NA^a^
Impaired body image (BIS score 21–30)	2 (6.2%)	NA^a^

*^a^NA, not applicable.*

Table 2 presents descriptive statistics and *t*-test comparisons.

**TABLE 2:** Descriptive statistics of individual and relational measures by role (patients vs. partners).

	Patients (*n* = 32)	Partners (*n* = 32)	Range	*T*-test	
				
Individual measures	M ± SD	M ± SD	Likert scale	t (df)	Sig.
Extraversion (BFQ-2)	4.60 ± 1.21	4.86 ± 1.08	1–7	−0.98 (62)	*p* = 0.367
Agreeableness (BFQ-2)	6.32 ± 0.65	5.56 ± 0.94		3.680 (62)	*p* = 0.0001**
Conscientiousness (BFQ-2)	5.55 ± 1.02	5.41 ± 1.10		0.509 (62)	*p* = 0.612
Openness (BFQ-2)	2.31 ± 1.01	3.25 ± 1.41		−3.104 (62)	*p* = 0.003*
Neuroticism (BFQ-2)	4.54 ± 1.20	5.09 ± 1.10		−1.935 (62)	*p* = 0.58
Forward focus (PACT)	5.64 ± 0.84	5.16 ± 0.75	1–7	2.385 (62)	*p* = 0.020*
Trauma focus (PACT)	4.92 ± 0.77	4.88 ± 0.95	1–7	0.205 (62)	*p* = 0.838
Flexibility (PACT)	0.84 ± 0.06	0.84 ± 0.05	0–1	−0.229 (62)	*p* = 0.820
Anxiety (HADS-A)	7.66 ± 4.95	6.47 ± 3.51	0–3	1.106 (62)	*p* = 0.273
Depression (HADS-D)	4.95 ± 2.65	3.87 ± 3.34	0–3	0.497 (62)	*p* = 0.621
Body Image (BIS)	6.47 ± 7.31	NA^a^	0–3	NA^a^	NA^a^
Fighting spirit (Mini-MAC)	2.94 ± 0.63	NA^a^	1–4	NA^a^	NA^a^
Helplessness-hopelessness (Mini-MAC)	1.67 ± 0.52	NA^a^		NA^a^	NA^a^
Fatalism (Mini-MAC)	2.66 ± 0.81	NA^a^		NA^a^	NA^a^
Anxious preoccupation (Mini-MAC)	2.19 ± 2.64	NA^a^		NA^a^	NA^a^
Avoidance (Mini-MAC)	2.39 ± 0.99	NA^a^		NA^a^	NA^a^
**Relational measures**					
Supportive dyadic coping_self (DCQ)	3.72 ± 0.70	3.5 ± 0.73	1–5	1.242 (62)	*p* = 0.219
Supportive dyadic coping_other (DCQ)	3.62 ± 0.82	3.63 ± 0.83		−0.30 (62)	*p* = 0.976
Delegating dyadic coping_self (DCQ)	3.00 ± 1.07	3.56 ± 0.82		−2.349 (62)	*p* = 0.022*
Delegating dyadic coping_other (DCQ)	3.35 ± 1.00	3.01 ± 0.89		1.442 (62)	*p* = 0.154
Common dyadic coping (DCQ)	3.74 ± 0.85	3.57 ± 0.72		0.879 (62)	*p* = 0.383
Negative dyadic coping_self (DCQ)	1.48 ± 0.46	1.73 ± 0.65		−1.811 (62)	*p* = 0.075
Negative dyadic coping_other (DCQ)	1.60 ± 0.65	1.63 ± 0.66		−0.227 (62)	*p* = 0.821
Stress communication_self (DCQ)	3.37 ± 0.86	2.78 ± 0.80		2,910 (62)	*p* = 0.007*
Stress communication_other (DCQ)	2.61 ± 1.01	3.50 ± 0.65		−4.134 (62)	*p* = 0.0001**
Coping evaluation (DCQ)	4.04 ± 0.90	3.75 ± 0.79		1.399 (62)	*p* = 0.167
Closeness (IOS)	5.56 ± 1.70	5.78 ± 1.28	1–7	−0.580 (62)	*p* = 0.564

*p < 0.05.

**p < 0.001.

*^a^NA, not applicable.*

### Cluster Analysis

Results of the cluster analysis supported our first hypothesis regarding variables affecting healthy vs. impaired functioning of patients and partners when coping with cancer. A 2-cluster solution was selected to discriminate among patients with statistically significant mean score variation by body image, anxious preoccupation, negative dyadic coping, and stress communication. Not all the variables were meaningful for the clusters, and significant F tests are summarized in Table 3. The first cluster (*n* = 24; 75%) included patients who experienced positive body image after surgery (M_BIS_ = 3), low levels of anxious preoccupation (M_Mini–MAC_ = 2.04), low levels of negative dyadic coping strategies, either self-(M_DCQ_ = 1.4) and other-reported (M_DCQ_ = 1.5), and lower stress communication rates self-related than patients in cluster two (M_DCQ_ = 3.19). According to these individual/relational variables, cluster 1 was labeled as: “*Active patients*,” indicating patients who actively adopt individual/relational resources, functional coping styles, and report elevated mood and self-rated wellbeing. The second cluster included patients (*n* = 8; 25%) with high levels of body image concerns (M_BIS_ = 17) and anxious preoccupation (M_Mini–MAC_ = 2.63), high levels of negative dyadic coping strategies about themselves (M_DCQ_ = 1.9) and the partner (M_DCQ_ = 2.0), and a greater use of stress communication within the couple (M_DCQ_ = 3.94). Patients’ cluster 2 was labeled “*Worried patients*,” indicating patients who fear the effects of therapies on their body appearance and show intense concerns facing individually the stressor, talking about it intensely within the couple, but without perceiving a functional dyadic coping in the partnership. No significant differences were detected for other variables considered.

**TABLE 3:** Cluster Analysis on patients’ subsample: ANOVA.

Measures	Cluster mean square	df	Error mean square	df	F	Sig.
Body Image (BIS)	1239.844	1	13.871	30	89.385	*p* = 0.0001**
Anxious Preoccupation (Mini-MAC)	2.078	1	0.346	30	6.001	*p* = 0.020*
Negative dyadic coping_self (DCQ)	1.450	1	0.175	30	8.275	*p* = 0.007*
Negative dyadic coping_other (DCQ)	1.707	1	0.380	30	4.486	*p* = 0.043*
Stress Communication_self (DCQ)	3.375	1	0.654	30	5.159	*p* = 0.030*

*p < 0.05 (two-tailed).

**p < 0.001 (two-tailed).

Then, the K-mean cluster analysis on partners’ scores resulted in a 2-cluster solution. Similarly, not all the variables were significantly different among the partners; significant *F* tests are summarized in Table 4. Partners in cluster 1 (*n* = 25; 78.1%) were characterized by anxiety (M_HADS–A_ = 5) and depression (M_HADS–D_ = 2) below of the clinical cut-offs (≥11), low scores on self-related negative dyadic coping style (M_DCQ_ = 1.6) and interpersonal closeness (M_IOS_ = 6), which enable them to maintain a sense of differentiation between the self and the other. This cluster was labeled as: “*Comfortable partners*,” indicating partners who demonstrate confidence in their role as caregivers. This was exemplified by stable mood and the ability to meet the needs of the dyad while maintaining a functional sense of differentiation from patients. Partners in cluster 2 (*n* = 7; 21.9%), showed “abnormal” levels of anxiety (M_HADS–A_ = 11), and “borderline” levels of depression (M_HADS–D_ = 9), elevated self-related negative dyadic coping (M_DCQ_ = 2.2), and inability to differentiate oneself from the partner (M_IOS_ = 7). Partners’ cluster two was labeled as “*Uncomfortable partners*,” indicating partners struggling to adjust the tasks and emotional responsibilities required by the caregiving function. Partners in this group showed a clearly compromised mood, poor ability to engage in functional dyadic coping strategies, and the inability of differentiating their experiences from the patients’. No significant differences were registered for the remaining measures.

**TABLE 4:** Cluster Analysis on partners’ subsample: ANOVA.

Measures	Cluster mean square	df	Error mean square	df	F	Sig.
Closeness (IOS)	7.800	1	1.456	30	89.385	*p* = 0.028*
Negative dyadic coping_self (DCQ)	1.687	1	0.382	30	4.413	*p* = 0.044*
Depression (HADS-D)	222.403	1	4.170	30	53.335	*p* = 0.0001**
Anxiety (HADS-A)	220.414	1	5.385	30	40.930	*p* = 0.0001**

*p < 0.05 (two-tailed).

**p < 0.001 (two-tailed).

### Dyadic Profiles

Three dyadic profiles emerged from this analysis: (1) *functional relationships* (*n* = 19 couples; 59.4%), with both partners belonging to cluster 1; (2) *dysfunctional relationships* (*n* = 2 couples; 6.2%), in which both partners reported impaired individual and relational wellbeing (cluster 2); and (3) *ambivalent relationships* (*n* = 11 couples; 34.4%) where each partner belonged to two different clusters. This result confirmed our hypothesis regarding couples’ functioning. However, it also revealed that there are groups of dyads characterized by lack of congruence in terms of coping and functioning in the cancer aftermath, as evidenced by stressful, incoherent, and oppositional responses. Statistically significant differences were reported for partner-perceived delegating dyadic coping (DCQ) [*F*_(2,61)_ = 4.838, *p* = 0.011], anxiety (HADS-A) [*F*_(2,61)_ = 5.049, *p* = 0.009], and depression (HADS-D) [*F*_(2,61)_ = 5.961, *p* = 0.004]. *Post hoc* tests with Bonferroni correction indicated that couples in functional relationships engaged more often in delegating strategies (M = 3.197; SD = 0.97), as compared to couples characterized by dysfunctional relationships (M = 1.89; SD = 1.18). Functional couples scored low on anxiety (M = 5.74; SD = 3.71) compared to ambivalent couples (M = 8.91; SD = 4.75), while they reported the lowest depression score (M = 3.13; SD = 2.17) when compared to both dysfunctional (M = 7.0; SD = 2.16) and ambivalent couples (M = 5.14; SD = 3.70).

## Discussion

The present contribution explored rates of body image concerns among BC patients and identified resulting dyadic profiles of couples facing the disease in the immediate post-operative period. Most patients experienced low levels of body image concerns, as anticipated by previous literature linking conservative surgery to better physical adjustment (Fingeret et al., 2013). Furthermore, patients were in their 60s and confirmed previous studies (e.g., Rosenberg et al., 2013; Champion et al., 2014; Leigh et al., 2019) that found lower concerns in older patients.

Despite the small sample size, the cluster analysis revealed two different clusters for patients (*active vs. worried*), and partners (*comfortable vs. uncomfortable*). Cluster 1 included individuals with better psychosocial and relational wellbeing. Women had low or absent body image concerns, compared to cluster two. Findings confirm the crucial role of body image, individual coping strategies (e.g., anxious preoccupation), stress communication, and negative dyadic coping in delineating different patterns of patients’ wellbeing. In line with theories that consider body image as structured by perceptive, emotional, and relational dimensions (White, 2000; Cash, 2004; Fingeret et al., 2014), patients and partners reported an overall elevated wellbeing. It is possible that patients reporting body image concerns also faced more challenges coping with the illness and perceived their partners to engage more often in negative dyadic behaviors. Further research is needed to investigate how the association between individual/relational variables and body image concerns evolves over time. Moreover, relational variables such as stress communication and negative dyadic coping may contribute to inform women’s physical and psychological adjustment. Similarly, partners significantly differed in their level of depression, anxiety, negative dyadic coping (self-perception), and interpersonal closeness. Facing breast cancer can create different configurations of emotional and relationship exchange among partners. In line with the existing literature (Maliski et al., 2002), common and positive dyadic coping strategies contributed to higher emotional and relational outcomes.

Three different dyadic profiles were identified (*functional, dysfunctional, and ambivalent relationships)*. It is important to note that anxiety, depression, and delegating dyadic coping significantly varied between couples. Several possible factors may contribute to the differences recorded between profiles. First, when women have low or absent levels of body image concerns and the partners rate their emotional and psychological wellbeing in a similar manner, they may be less anxious and depressed. Second, positive dyadic coping (i.e., delegated) characterized couples better adjusted to the illness. When couples are committed to mutual support, they feel they can rely on their partner’s resources and they are more engaged to achieve common goals; an ability which leads to the perception of greater effectiveness to cope and overcome the stressful event (Brandão et al., 2017). Finally, delegated dyadic coping involves efforts to help the partner reducing stress by taking over some tasks and responsibilities (Keesing et al., 2016; Falconier and Kuhn, 2019). According to the *Systemic-Transactional Model of Dyadic Coping* (Bodenmann, 1995), positive dyadic coping strategies benefit both partners’ psychological and relationship functioning. Overall, our results are consistent with previous studies examining short and long-term consequences of cancer, especially in relation to marital satisfaction (Hagedoorn et al., 2008; Dekel et al., 2014). In the last 30 years, the application of a dyadic framework has identified variables able to increase wellbeing and satisfaction, since cancer has negative implications for both (Scott et al., 2004; Stanton et al., 2007; Kim et al., 2016; Saita et al., 2016). Several studies have illustrated the significant association between self-reported dyadic coping and partner’s outcomes, and the importance of the congruence between partners’ coping strategies (Kayser et al., 2007).

Some limitations should be discussed. First, to answer the research question, and unable to recruit further dyads for the above-mentioned reasons, we used the k-mean cluster analysis with a limited sample size (Dagan and Hagedoorn, 2014). This could affect the generalization of results to other groups. A more properly powered sample is needed to investigate the different profiles of couples dealing with breast cancer. Second, the decision to collect data from a single institution might affect external validity of the findings. Future research is needed to recruit larger and more representative samples. Third, patients varied in terms of cancer stage, type of surgery, and age. It would therefore be appropriate to investigate whether these variables influence participants’ wellbeing and stratify samples accordingly. Despite these limitations, the implementation of a relational approach allowed the research team to describe the psychological experience of both couple’s members and to explore dyadic profiles of relationship functioning 1 week after surgery. Future studies should investigate the role of relational variables on body image concerns among diverse cancer types and sexual minority survivors. It is recommended to add a qualitative exploratory phase to better understand couples’ experiences. It would also be relevant to examine the role of underlying interpersonal processes, as younger couples are characterized by unique psychosocial issues (Kayser and Acquati, 2019). Finally, we recommend exploring the role of individual and relational characteristics in the context of other diseases (e.g., Riazuelo, 2021; Weitkamp et al., 2021).

## Conclusion

Present results can help health care teams develop dyadic psychosocial interventions openly addressing body image concerns, in order to improve the quality of life and wellbeing of couples facing BC. By gaining an in-depth understanding of the mechanisms that inform behavior at the individual and couple level, it will be possible to assist researchers and clinicians in the field. Our findings, albeit preliminary, further confirm that the presence of a supportive partner contributes to women’s outcomes and that the interaction between partners can affect their relational wellbeing. For couples most at risk, such as those in the dysfunctional and ambivalent clusters, clinicians should focus on improving communication and dyadic coping skills to manage cancer-related stress.

## Author’s Note

An earlier version of this work was presented at the 20^th^ World Congress of the International Psycho-Oncology Society, November 2018, Hong Kong, with the title *Dyadic Profiles of Couples Coping with Breast Cancer: Results of a Cluster Analysis of Body Image Concerns.*

## Data Availability Statement

The raw data supporting the conclusions of this article will be made available by the authors, without undue reservation.

## Ethics Statement

The studies involving human participants were reviewed and approved by Ethic Commission for Psychology Research (CERPS), Department of Psychology, Catholic University of Sacred Heart of Milan. The patients/participants provided their written informed consent to participate in this study.

## Author Contributions

ES and CA contributed to the conception, design, and methodology of the study and wrote and revised sections of the manuscript. GF and DV contributed to the data collection, performed statistical analysis, and wrote and revised the first draft of the manuscript. SM and AS contributed to the methodology and wrote and revised sections of the manuscript. FV and MG contributed to the data collection, the organization of database, and the revision of the manuscript. All authors contributed to manuscript revision, read, and approved the submitted version.

## Conflict of Interest

The authors declare that the research was conducted in the absence of any commercial or financial relationships that could be construed as a potential conflict of interest.

## Publisher’s Note

All claims expressed in this article are solely those of the authors and do not necessarily represent those of their affiliated organizations, or those of the publisher, the editors and the reviewers. Any product that may be evaluated in this article, or claim that may be made by its manufacturer, is not guaranteed or endorsed by the publisher.
